# Potential of Apigenin, Berberine, Chrysin, and Luteolin to Overcome Doxorubicin Resistance in Acute Promyelocytic Leukemia HL-60 Cells

**DOI:** 10.3390/ijms262110565

**Published:** 2025-10-30

**Authors:** Piotr Wadowski, Katarzyna Woźniak

**Affiliations:** 1 Department of Molecular Genetics, Faculty of Biology and Environmental Protection, University of Lodz, Pomorska 141/143, 90-236 Lodz, Poland; piotr.wadowski@edu.uni.lodz.pl; 2 Doctoral School of Exact and Natural Sciences, University of Lodz, Banacha 12/16, 90-237 Lodz, Poland

**Keywords:** doxorubicin, resistance, Apigenin, Berberine, Chrysin, Luteolin, HL-60 cells, NRF2 inhibitors

## Abstract

Multidrug resistance (MDR) is one of the leading causes of high mortality in cancer. The NRF2 (Nuclear Factor Erythroid 2-Related Factor 2) transcriptional factor can play a major role in MDR development. In this study, we tested the NRF2 inhibitors—Apigenin, Berberine, Chrysin, and Luteolin (ABCL) for their ability to overcome doxorubicin resistance in acute promyelocytic leukemia HL-60 cells (HL-60/DOXO cells). We examined the effects of NRF2 inhibitors on cell viability, apoptosis, DNA damage, and reactive oxygen species (ROS) generation. Our results indicate that Apigenin, Chrysin, and Luteolin can effectively overcome doxorubicin resistance in HL-60/DOXO cells. On the contrary, Berberine does not demonstrate this activity and even hinders doxorubicin’s activity. We hypothesize that the observed effects of ABCL may result from their interaction with the NRF2 factor, which is hyperactivated in the tested HL-60/DOXO cells.

## 1. Introduction

In 2020, leukemia was the 15th most commonly occurring cancer and the 6th most lethal cancer worldwide [1]. In 2024, it was estimated that over 60,000 new cases of leukemia had occurred, with the disease itself resulting in the deaths of over 23,000 individuals in the United States alone [2]. Leukemia can be classified using two criteria—rapidity of proliferation or maintenance of cell differentiation (acute or chronic) and origin (myeloid or lymphoid)—that are into four major types: acute myeloid leukemia (AML), acute lymphocytic leukemia (ALL), chronic myeloid leukemia (CML), and chronic lymphocytic leukemia (CLL) [3]. AML is the most common type of leukemia and accounts for about 80% and 30% of all cases in adults and children, respectively [4]. Acute promyelocytic leukemia (APL) is a subtype of AML that makes up about 15% of all AML cases [5]. A characteristic feature of APL is the t(15;17) reciprocal translocation, which is present in about 90% of all APL cases, as this translocation involves the fusion of retinoic acid receptor alpha (RARα) from chromosome 17 and promyelocytic leukemia gene from chromosome 15 [5,6]. This results in the production of PML-RARα oncoprotein and subsequent blockage of differentiation of the promyelocytes [6]. The HL-60 cell line serves as an example of APL. The HL-60 cell line piques particular interest in research due to the specific mutations of c-myc, *p53*, and *N-ras* oncogenes, as well as its ability to differentiate into various myelomonocytic cells in vitro, whilst these cells are usually lacking in some of the characteristics of the corresponding normal cells [7].

**Figure 1:**
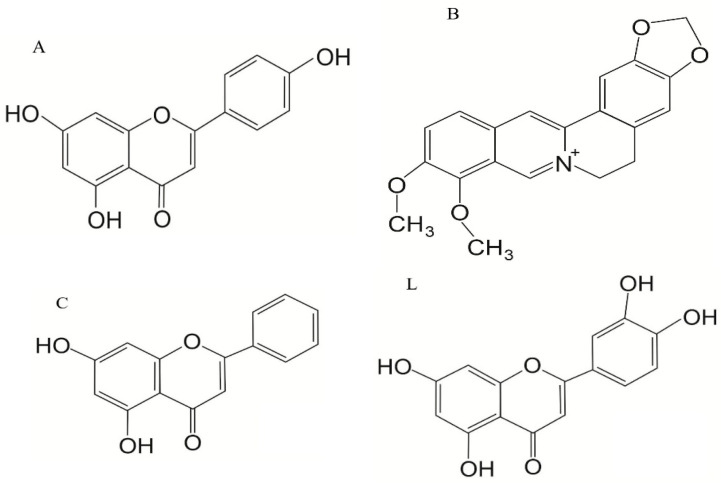
Chemical structure of the ABCL: Apigenin (A), Berberine (B), Chrysin (C), and Luteolin (L).

The most common chemotherapy regimen in AML is treatment with cytarabine, often combined with anthracyclines, such as doxorubicin (DOXO) (also known as Adriamycin/ADM) or, more frequently, daunorubicin, as a first-line treatment [8,9,10]. DOXO is an anthracycline-based anti-cancer drug that intercalates into DNA, induces oxidative stress, inhibits the activity of topoisomerase II, and disrupts mitochondrial function. Another noteworthy feature of DOXO is that it triggers CD8+ T-cell responses, which boost anti-cancer responses of the immune system [11]. However, just like many other chemotherapeutics, its effectiveness is heavily affected by the development of multidrug resistance (MDR) in cancer cells. MDR can be described as cancer developing resistance to a wide range of anti-cancer therapeutics, which are functionally and structurally different when compared to the drug that was initially being used in that particular cancer’s therapy [12]. It can be acquired through a variety of ways, one of which includes hyperactivation of the NRF2 transcriptional factor (Nuclear Factor Erythroid 2-Related Factor 2) [13]. NRF2 is considered to be the cell’s main protector against oxidative stress, as it regulates the expression of numerous genes, including those responsible for metabolism of xenobiotics, response to oxidative stress, inflammation, and mitochondrial function. And it is this regulatory function that helps MDR cancers with upregulated NRF2 to overcome the treatment. It is for that reason that this particular transcriptional factor may prove to be a perfect target for both improving the effectiveness of anti-cancer treatment as well as surmounting ever-present MDR. The use of DOXO is also limited due to its widely described cardiotoxicity, which is caused mainly by the induction of the production of excessive amounts of reactive oxygen and nitrogen species [3,11]. DOXO resistance is mainly acquired through signaling pathways, which promote cell cycle progression and replication and prevent autophagic and apoptotic cell death, in addition to promoting overexpression and upregulation of ATP membrane transporters, in various proteins, epithelial–mesenchymal transition, and increased DNA repairs, as well as post-translational modifications. DOXO is often used in the therapy of other cancers, such as breast and lung cancers, myelomas, and soft tissue sarcomas [14]. As more attention is being drawn towards methods for overcoming MDR in cancer, the search is on for NRF2 inhibitors, both synthetic and natural. While synthetic compounds are being developed, and some have even proven to be quite effective like ML385 [15,16], K67 [16] AEM1 [17], MSU38225 [18], and pyrazolyl hydroxamic acid derivatives [19], the overall task proves to be challenging. And thus, the interest has shifted towards compounds of natural origin, of which a lot have been proven to have an inhibitory effect on NRF2 [20]. We picked Apigenin (A), Berberine (B), Chrysin (C), and Luteolin (L) (later described as ABCL) to test their ability to overcome doxorubicin resistance in HL-60 cells, as their ability to inhibit the activity of NRF2 through decreasing levels of its protein and mRNA has been widely documented [20]. Chemical structures of the ABCL are shown in Figure 1.

## 2. Results and Discussion

### 2.1. ABCL Cytotoxicity

**Figure 2:**
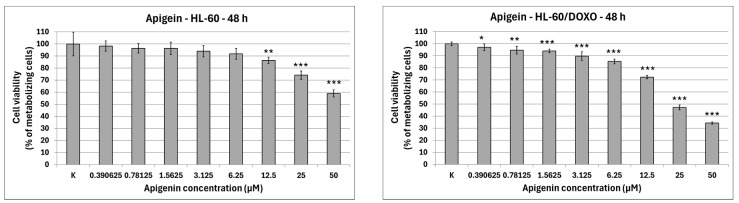

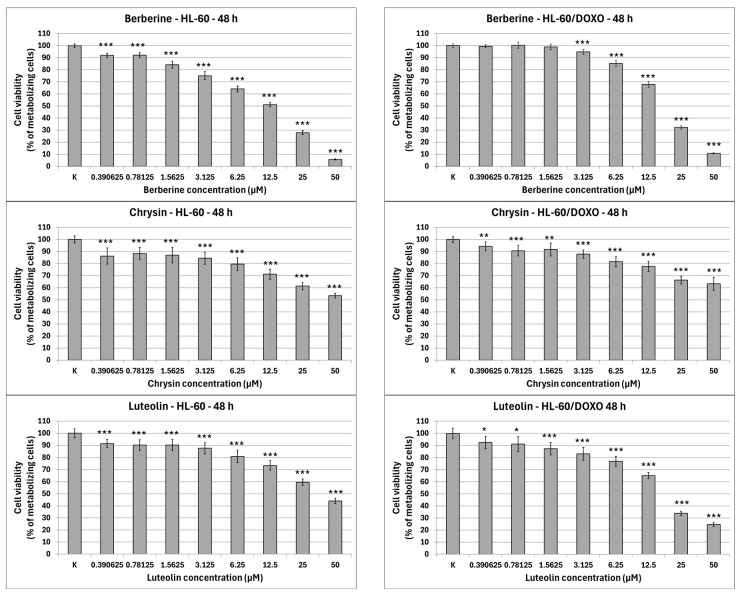
ABCL cytotoxicity after 48 h incubation in HL-60 and HL-60/DOXO cells. Results are shown as mean percentage ± SD, * *p* < 0.05, ** *p* < 0.01, and *** *p* < 0.001, compared to the control (K).

Apigenin is moderately cytotoxic towards doxorubicin-sensitive HL-60 cells with an IC_50_ at 40.15 µM, significantly reducing the percentage of metabolizing cells to 86.42 ± 2.56% at 12.5 µM (*p* < 0.01), 74.11 ± 3.50% at 25 µM (*p* < 0.001), and 58.97 ± 2.98% at 50 µM (*p* < 0.001), while exhibiting higher cytotoxicity towards doxorubicin-resistant HL-60/DOXO cells, with an IC_50_ at 17.26 µM, significantly reducing the percentage of metabolizing cells already from the smallest concentration (Figure 2). Berberine is highly cytotoxic towards both doxorubicin-sensitive cells, with an IC_50_ at 16.66 µM, significantly reducing the percentage of metabolizing cells. At the highest concentration used (50 µM), Berberine reduces the number of living cells to 5.74 ± 0.39% in HL-60 cells (*p* < 0.001) and to 10.77 ± 0.49% in HL-60/DOXO cells (*p* < 0.001), with an IC_50_ at 18.42 µM. Chrysin is moderately cytotoxic towards both HL-60 and HL-60/DOXO cells, reducing the percentage of metabolizing cells at all used concentrations. Chrysin is also the least cytotoxic out of ABCL, with an IC_50_ at 90.26 µM and 102.58 µM for HL-60 and HL-60/DOXO, respectively. Luteolin is moderately cytotoxic towards doxorubicin-sensitive HL-60 cells, with an IC_50_ at 43.41 µM, significantly reducing the percentage of metabolizing cells at all used concentrations. At the highest concentration used (50 µM), Luteolin reduces the number of living cells to 44.14 ± 2.10% (*p* < 0.001). Luteolin exhibits higher cytotoxicity towards doxorubicin-resistant HL-60/DOXO cells, with an IC_50_ at 17.81 µM, reducing the percentage of metabolizing cells to 24.69 ± 1.70 at 50 µM (*p* < 0.001).

The results of 24 h incubation with ABCL are included in the Supplementary Materials (Figure S1); overall, the effects of ABCL on both sensitive and resistant cell lines were similar to 48 h incubation, with the key difference being lower cytotoxicity due to shorter exposure time. As with the 48 h incubation, Berberine was found to be the most cytotoxic, while Chrysin was found to be the least cytotoxic (Figure S1).

### 2.2. Choice of Incubation Model—48 h Preincubation with ABCL and Doxorubicin 24 h Incubation

**Figure 3:**
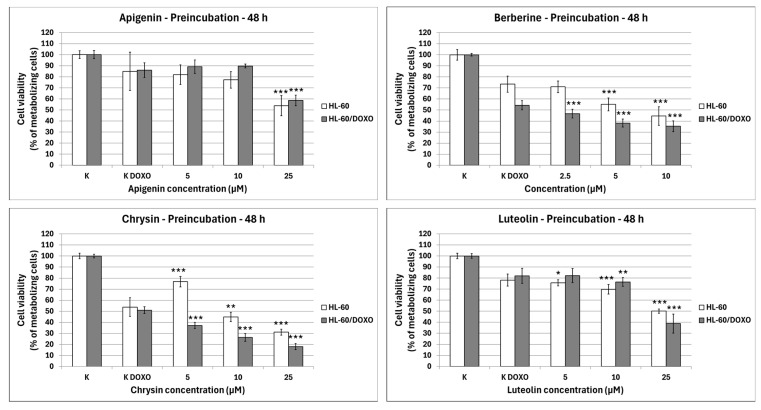
Doxorubicin cytotoxicity after 48 h preincubation with ABCL followed by 24 h incubation with doxorubicin. Results are shown as mean percentage ± SD, * *p* < 0.05, ** *p* < 0.01, and *** *p* < 0.001, compared to the doxorubicin control (K DOXO).

After 48 h preincubation with ABCL and a subsequent 24 h incubation with doxorubicin, Apigenin reduced the percentage of metabolizing cells to 53.89 ± 9.20% at 25 µM concentration (*p* < 0.001) compared to 84.97 ± 17.21% in doxorubicin control in doxorubicin-sensitive HL-60 cells, and 58.62 ± 4.86% at 25 µM concentration (*p* < 0.001) compared to 86.05 ± 6.64% in doxorubicin control in doxorubicin-resistant HL-60/DOXO cells (Figure 3). Berberine reduced the percentage of metabolizing cells to 55.14 ± 5.80% at 5 µM (*p* < 0.001) and 44.54 ± 8.36% at 10 µM concentration (*p* < 0.001) compared to 73.42 ± 7.25% in doxorubicin control in doxorubicin-sensitive cell line, and 46.76 ± 3.90% at 2.5 µM (*p* < 0.001), 38.21 ± 3.57% at 5 µM (*p* < 0.001) and 35.30 ± 4.90% at 10 µM concentration (*p* < 0.001), compared to 54.32 ± 4.12% in doxorubicin control in doxorubicin-resistant cell line. Chrysin increased the percentage of metabolizing cells to 76.78 ± 4.74% at 5 µM concentration (*p* < 0.001) but reduced it to 44.91 ± 4.26% at 10 µM (*p* < 0.01) and 31.13 ± 2.67% at 25 µM concentration (*p* < 0.001) compared to 53.83 ± 8.43% in the doxorubicin control in the doxorubicin-sensitive cell line. In the doxorubicin-resistant cell line, Chrysin reduced the percentage of metabolizing cells to 37.18 ± 2.94% at 5 µM (*p* < 0.001), 26.30 ± 3.53% at 10 µM (*p* < 0.001), and 17.99 ± 2.77% at 25 µM concentration (*p* < 0.001) compared to 50.99 ± 3.05% in the doxorubicin control. Luteolin reduced the percentage of metabolizing cells to 75.74 ± 2.79% at 5 µM (*p* < 0.05), 69.80 ± 4.35% at 10 µM (*p* < 0.001), and 50.07 ± 1.86% at 25 µM concentration (*p* < 0.001) compared to 78.11 ± 5.46% in doxorubicin control in doxorubicin-sensitive cell line, and 76.46 ± 3.96% at 10 µM (*p* < 0.01) and 38.82 ± 8.62% at 25 µM concentration (*p* < 0.001) compared to 82.01 ± 6.80% in doxorubicin control in doxorubicin-resistant cell line.

Because the 48 h preincubation model with ABCL has been shown to exhibit the highest increase in effectiveness of doxorubicin-induced cytotoxicity when compared to 24 h coincubation as well as 24 h preincubation, this model was chosen for all further experiments (results of other incubation models with ABCL are included in the Supplementary Materials, Figures S2 and S3). Additionally, centrifugation after incubation with ABCL ensured that cells that died due to ABCL’s cytotoxicity were separated at that point.

Gao et al. have shown that Apigenin sensitizes BEL-7402/ADM cells to doxorubicin after 48 h preincubation followed by 48 h incubation with doxorubicin [21]. Paramasivan et al. acquired similar results with neferine in A549 and A549/DOXO cell lines, and also observed the increased effectiveness of doxorubicin when combined with neferine, when compared to doxorubicin alone [22].

### 2.3. DNA Damage

**Figure 4:**
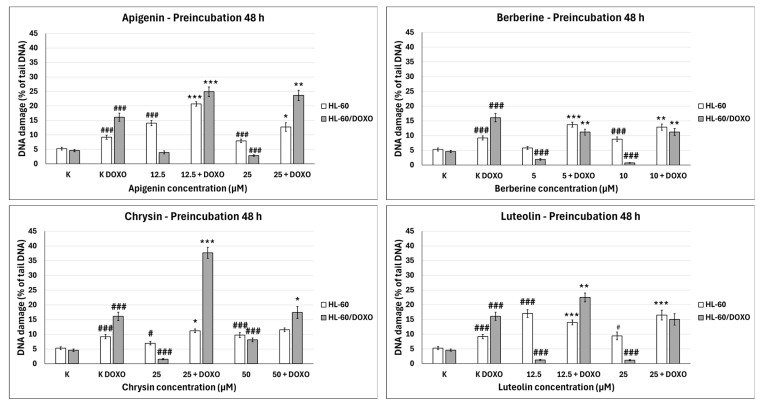
Doxorubicin-induced DNA damage after 48 h preincubation with ABCL followed by 24 h incubation with doxorubicin. Results are shown as mean percentage ± SEM, # *p* < 0.05, ### *p* < 0.001, compared to the control (K), * *p* < 0.05, ** *p* < 0.01, and *** *p* < 0.001, compared to the doxorubicin control (K DOXO).

Apigenin increased the DNA damage percentage in doxorubicin-sensitive HL-60 cells to 14.06 ± 0.92% at 12.5 µM (*p* < 0.001) and 7.95 ± 5.90% at 25 µM (*p* < 0.001) compared to 5.28 ± 0.50% in control (Figure 4). In combination with doxorubicin, the DNA damage percentage increased to 20.67 ± 0.78% at 12.5 µM (*p* < 0.001) and 12.73 ± 1.47% at 25 µM (*p* < 0.05) compared to 9.19 ± 0.72% in the doxorubicin control. Apigenin reduced the DNA damage percentage in doxorubicin-resistant HL-60/DOXO cells to 3.93 ± 0.52% at 25 µM (*p* < 0.001) compared to 4.59 ± 0.40% in control. In combination with doxorubicin, the DNA damage percentage increased to 24.91 ± 1.67 at 12.5 µM (*p* < 0.001) and 23.64 ± 1.81 at 25 µM (*p* < 0.01) compared to 16.09 ± 1.40 in the doxorubicin control.

Berberine increased the DNA damage percentage in doxorubicin-sensitive HL-60 cells to 8.77 ± 0.75% at 10 µM (*p* < 0.001) compared to 5.28 ± 0.50% in control; in combination with doxorubicin, the DNA damage percentage increased to 13.70 ± 0.83% at 5 µM (*p* < 0.001) and 12.85 ± 1.05% at 10 µM (*p* < 0.01) compared to 9.19 ± 0.72% in the doxorubicin control. Berberine decreased the DNA damage percentage in doxorubicin-resistant HL-60/DOXO cells to 1.93 ± 0.26% at 5 µM (*p* < 0.001) and 0.71 ± 0.14% at 10 µM (*p* < 0.001) compared to 4.59 ± 0.40% in the control. In combination with doxorubicin, the DNA damage percentage decreased to 11.19 ± 1.00% at 5 µM (*p* < 0.01) and 11.19 ± 1.18% at 10 µM (*p* < 0.01) compared to 16.09 ± 1.40% in the doxorubicin control.

Chrysin increased the DNA damage percentage in doxorubicin-sensitive HL-60 cells to 6.98 ± 0.59% at 25 µM (*p* < 0.05) and 9.72 ± 0.86% at 50 µM (*p* < 0.001) compared to 5.28 ± 0.50% in control; in combination with doxorubicin, the DNA damage increased to 11.17 ± 0.62% at 25 µM (*p* < 0.05) compared to 9.19 ± 0.72% in the doxorubicin control. Chrysin decreased the DNA damage percentage to 1.56 ± 0.21% at 25 µM (*p* < 0.001) and increased the DNA damage percentage to 8.12 ± 0.64% at 50 µM compared to 4.59 ± 0.40% in control in HL-60/DOXO cells. In combination with doxorubicin, the DNA damage percentage increased to 37.71 ± 1.87% at 25 µM (*p* < 0.001) and 17.40 ± 2.04% at 50 µM (*p* < 0.05) compared to 16.09 ± 1.40% in the doxorubicin control.

Luteolin increased the DNA damage percentage in doxorubicin-sensitive HL-60 cells to 17.04 ± 1.35% at 12.5 µM (*p* < 0.001) and 9.40 ± 1.32% at 25 µM (*p* < 0.05) compared to 5.28 ± 0.50% in control; in combination with doxorubicin, the DNA damage percentage increased to 14.01 ± 0.81% at 12.5 µM (*p* < 0.001) and 16.50 ± 1.64% at 25 µM (*p* < 0.001) compared to 9.19 ± 0.72% in the doxorubicin control. Luteolin reduced the DNA damage percentage in doxorubicin-resistant HL-60/DOXO cells to 1.27 ± 0.19% at 12.5 µM (*p* < 0.001) and 1.16 ± 0.19% at 25 µM (*p* < 0.001) compared to 4.59 ± 0.40% in the control. In combination with doxorubicin, the DNA damage percentage increased to 22.50 ± 1.52% at 12.5 µM (*p* < 0.01) compared to 16.09 ± 1.40% in the doxorubicin control.

**Figure 5:**
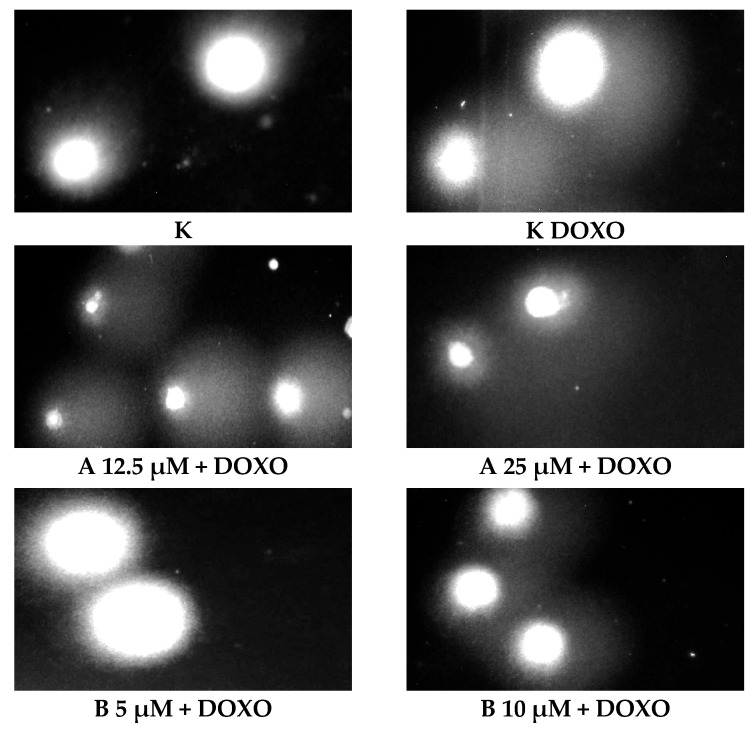

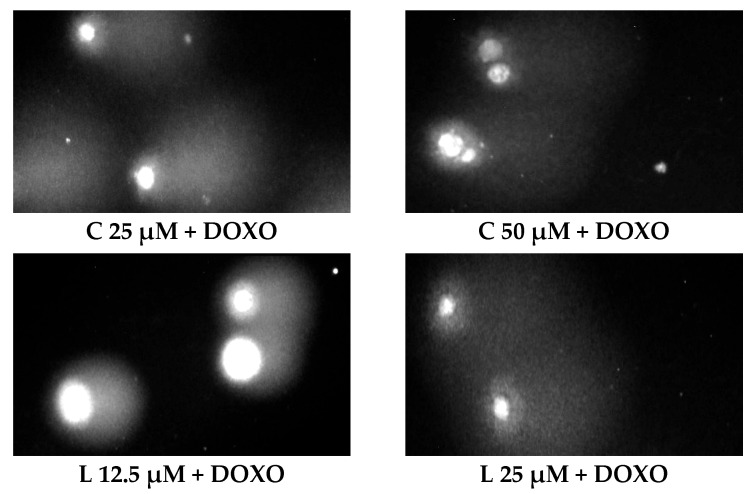
Representative pictures of comets obtained from HL-60/DOXO cells after 48 h preincubation with ABCL followed by 24 h incubation with doxorubicin. A—Apigenin; B—Berberine; C—Chrysin; L—Luteolin; DOXO—doxorubicin; K—control.

ABCL has shown the ability to induce DNA damage in doxorubicin-sensitive HL-60 cells; in combination with doxorubicin, it increases its ability to induce DNA damage. In doxorubicin-resistant HL-60/DOXO cells, ABCL has been shown to decrease DNA damage, while at the same time highly increasing the DNA damage-inducing properties of doxorubicin, one exception being Berberine, in which the combination with doxorubicin resulted in lower levels of DNA damage. Figure 5 shows representative comet images obtained after 48 h of preincubation of HL-60/DOXO cells with ABCL followed by 24 h of incubation with doxorubicin. The comet images correspond well with the numerical data describing the level of DNA damage presented in Figure 4. It should also be noted that Chrysin at 50 µM might have induced far greater DNA damage levels than shown in Figure 4; upon examination of photos of the sample in Figure 5, it is quite possible that the DNA damage was so high that it rendered accurate measurement impossible. Another noteworthy aspect is that in doxorubicin-sensitive cells, Apigenin has induced lower DNA damage at 25 µM than at 12.5 µM, and similar results were obtained by Arango et al. [23]; in their study, Apigenin at 50 µM concentration induced higher DNA damage levels after 1 and 3 h of incubation and lower DNA damage levels after 6 and 9 h of incubation. We suspect that this effect might be caused by the cells’ activation of DNA repair pathways caused by prolonged exposure to high concentrations of Apigenin.

### 2.4. Apoptosis

**Figure 6:**
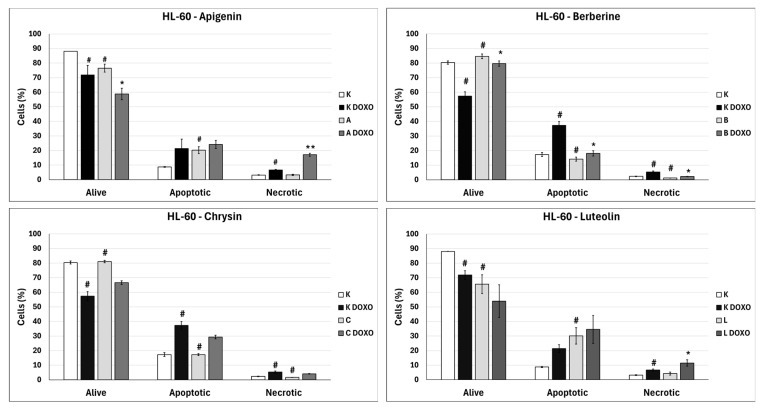
Doxorubicin-induced apoptosis in HL-60 cells after 48 h preincubation with ABCL followed by 24 h incubation with doxorubicin. A—Apigenin; A + DOXO—Apigenin + doxorubicin; B—Berberine; B + DOXO—Berberine + doxorubicin; C—Chrysin; C + DOXO—Chrysin + doxorubicin; L—Luteolin; L + DOXO—Luteolin + doxorubicin. Results are shown as mean percentage ± SD, # *p* < 0.05, compared to the control (K), * *p* < 0.05, ** *p* < 0.01, compared to the doxorubicin control (K DOXO).

**Figure 7:**
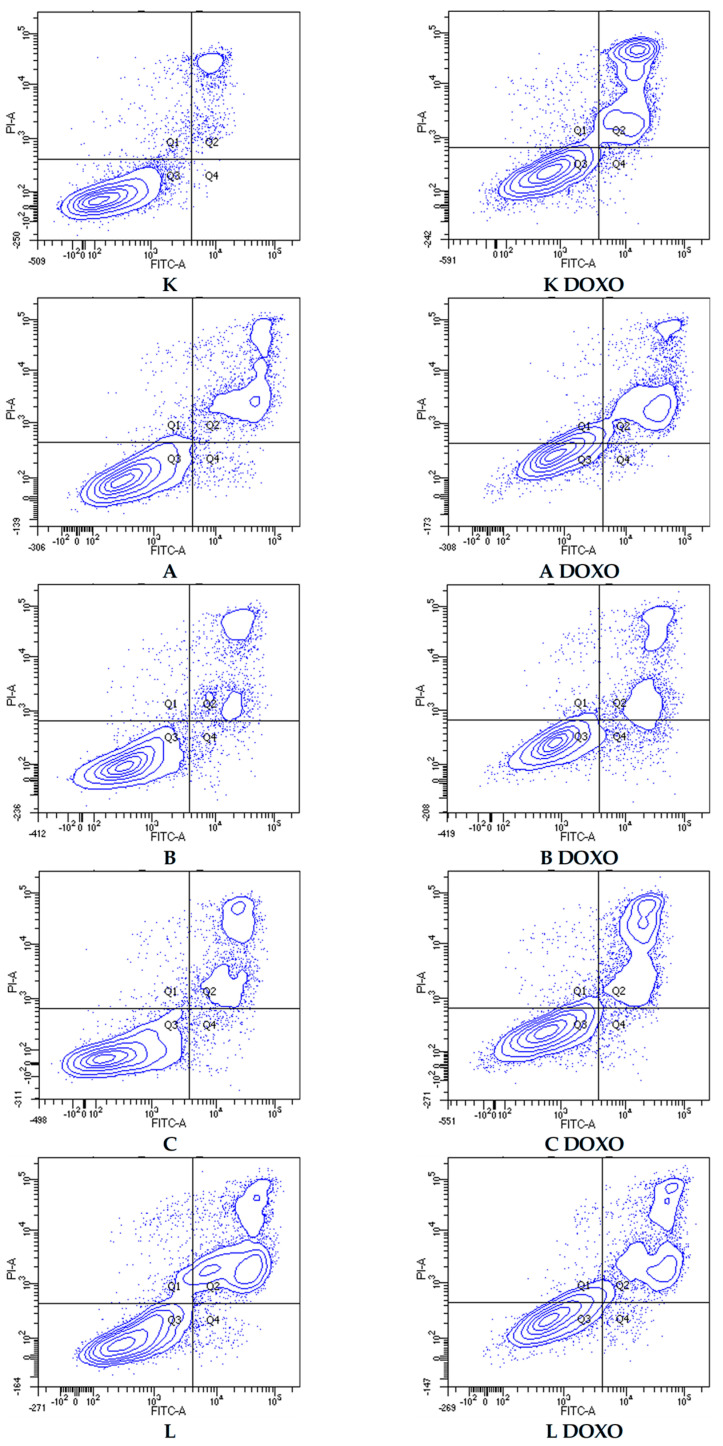
Representative flow cytometric dot plots of HL-60 cells after 48 h preincubation with ABCL followed by 24 h incubation with doxorubicin. A—Apigenin, A + DOXO—Apigenin + doxorubicin, B—Berberine, B + DOXO—Berberine + doxorubicin, C—Chrysin, C + DOXO—Chrysin + doxorubicin, L—Luteolin, and L + DOXO—Luteolin + doxorubicin. Q1—necrotic cells, Q2—late apoptosis, Q3—alive cells, and Q4—early apoptosis.

**Figure 8:**
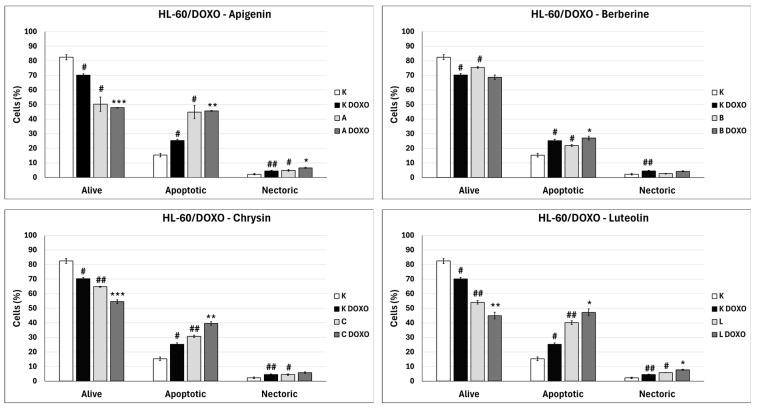
Doxorubicin-induced apoptosis in HL-60/DOXO cells after 48 h preincubation with ABCL followed by 24 h incubation with doxorubicin. A—Apigenin, A + DOXO—Apigenin + doxorubicin, B—Berberine, B + DOXO—Berberine + doxorubicin, C—Chrysin, C + DOXO—Chrysin + doxorubicin, L—Luteolin, L + DOXO—Luteolin + doxorubicin. Results are shown as mean percentage ± SD, # *p* < 0.05, and ## *p* < 0.01, compared to the respective control (K), * *p* < 0.05, ** *p* < 0.01, and *** *p* < 0.001, compared to the respective doxorubicin control (K DOXO).

In doxorubicin-sensitive HL-60 cells, incubation with Apigenin increased the percentage of apoptotic cells to 20.21 ± 2.43% (*p* < 0.05) compared to 8.74 ± 0.38% in control; in combination with doxorubicin, the percentage of necrotic cells increased to 17.09 ± 1.07% (*p* < 0.01) compared to 6.72 ± 2.69% in the doxorubicin control (Figure 6). Figure 7 shows representative flow cytometric dot plots of HL-60 cells after 48 h preincubation with ABCL followed by 24 h incubation with doxorubicin in viable (Q3), early apoptotic (Q4), late apoptotic (Q2), and necrotic (Q1) stages. In doxorubicin-resistant HL-60/DOXO cells, incubation with Apigenin increased the percentage of apoptotic cells to 44.86 ± 4.60% (*p* < 0.05) compared to 15.30 ± 1.24% in control; in combination with doxorubicin, the percentage of apoptotic cells increased to 45.64 ± 0.26% (*p* < 0.01) compared to 25.31 ± 0.88% in the doxorubicin control (Figure 8).

In doxorubicin-sensitive HL-60 cells after incubation with Berberine in combination with doxorubicin, the percentage of apoptotic cells decreased to 18.10 ± 1.77% (*p* < 0.05) compared to 37.32 ± 2.62% in the doxorubicin control (Figure 6). In doxorubicin-resistant HL-60/DOXO cells, incubation with Berberine increased the percentage of apoptotic cells to 21.94 ± 0.63% (*p* < 0.05) compared to 15.30 ± 1.24% in the control; in combination with doxorubicin, the percentage of apoptotic cells increased to 27.01 ± 0.72% compared to 25.31 ± 0.88% (*p* < 0.05) in the doxorubicin control (Figure 8).

In doxorubicin-sensitive HL-60 cells after incubation with Chrysin in combination with doxorubicin, no statistically significant differences were observed when compared to the doxorubicin control (*p* > 0.05) (Figure 6). In doxorubicin-resistant HL-60/DOXO cells, incubation with Chrysin increased the percentage of apoptotic cells to 30.79 ± 0.72% (*p* < 0.01) compared to 15.30 ± 1.24% in the control; in combination with doxorubicin, the percentage of apoptotic cells increased to 39.59 ± 1.30% (*p* < 0.01) compared to 25.31 ± 0.88% in the doxorubicin control (Figure 8).

In doxorubicin-sensitive HL-60 cells, incubation with Luteolin increased the percentage of apoptotic cells to 30.11 ± 5.56% (*p* < 0.05) compared to 8.74 ± 0.38% in control; in combination with doxorubicin, no statistically significant differences were observed when compared to the doxorubicin control (Figure 6). In doxorubicin-resistant HL-60/DOXO cells, incubation with Luteolin increased the percentage of apoptotic cells 40.12 ± 1.24% (*p* < 0.01) compared to 15.30 ± 1.24% in the control. In combination with doxorubicin, the percentage of apoptotic cells increased to 47.21 ± 2.36% (*p* < 0.05) compared to 25.31 ± 0.88% in the doxorubicin control (Figure 8).

In doxorubicin-sensitive HL-60 cells, ABCL increases the percentage of apoptotic cells; however, in combination with doxorubicin, no increase in apoptotic cell percentage is observed; furthermore, Berberine has caused a significant drop in apoptotic cells in combination with doxorubicin. Interestingly, Apigenin in combination with doxorubicin has been shown to greatly increase the percentage of necrotic cells (*p* < 0.01) (Figure 6).

**Figure 9:**
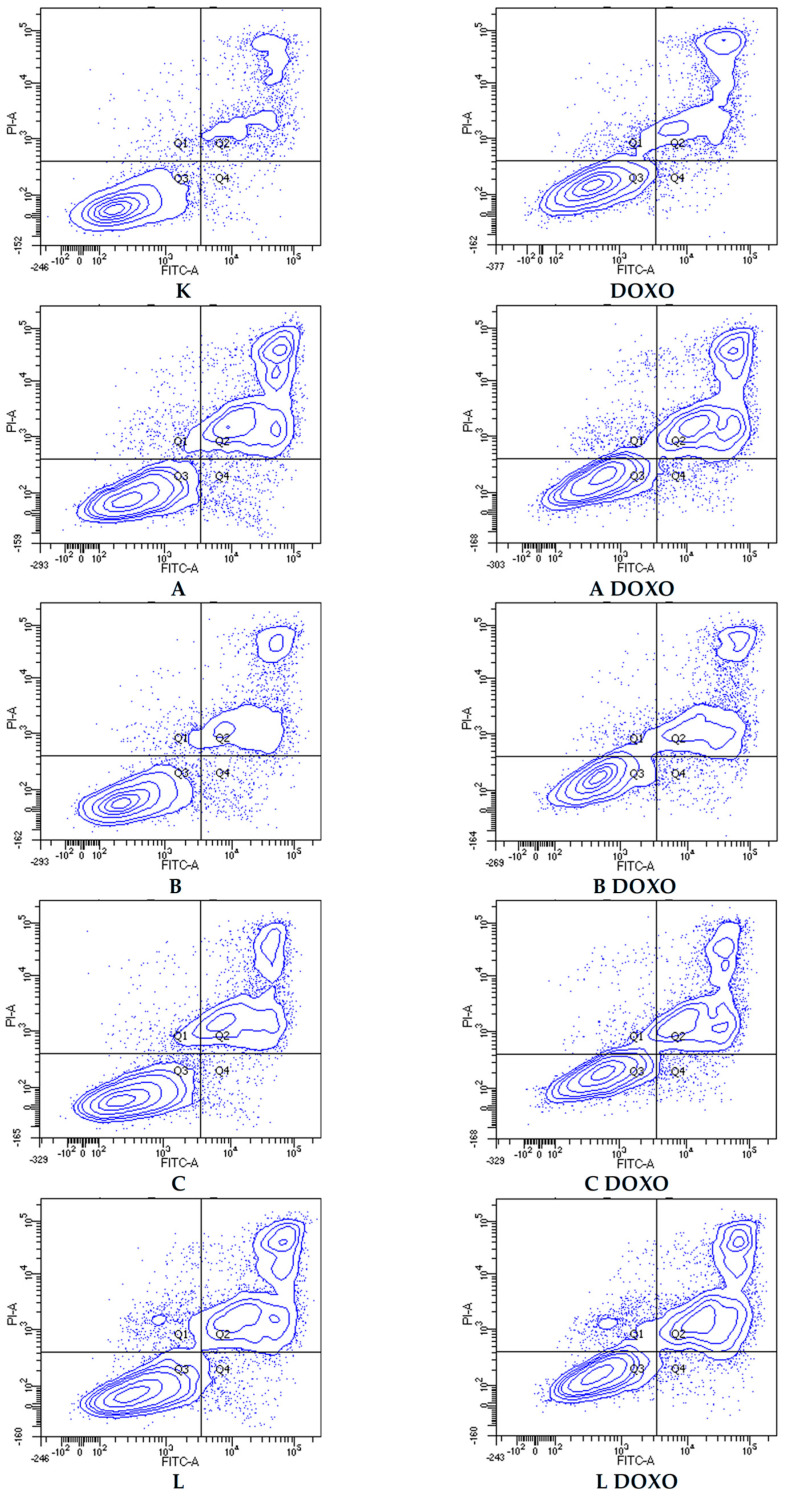
Representative flow cytometric dot plots of HL-60/DOXO cells after 48 h preincubation with ABCL followed by 24 h incubation with doxorubicin. A—Apigenin, A + DOXO—Apigenin + doxorubicin, B—Berberine, B + DOXO—Berberine + doxorubicin, C—Chrysin, C + DOXO—Chrysin + doxorubicin, L—Luteolin, and L + DOXO—Luteolin + doxorubicin. Q1—necrotic cells, Q2—late apoptosis, Q3—alive cells, and Q4—early apoptosis.

In doxorubicin-resistant HL-60/DOXO cells, ABCL increases the percentage of apoptotic cells. A combination of ABCL with doxorubicin also results in an increased percentage of apoptotic cells. It should be noted, however, that in cases of Apigenin and Berberine, this effect may be attributed to the compounds themselves (Figure 8). Figure 9 shows representative flow cytometric dot plots of HL-60/DOXO cells after 48 h preincubation with ABCL, followed by 24 h incubation with doxorubicin in viable (Q3), early apoptotic (Q4), late apoptotic (Q2), and necrotic (Q1) stages.

Paramasivan et al. showed that combined treatment of A549 and A549/DOXO cells with doxorubicin and neferine for 48 h increased the percentage of phosphatidyl serine externalization and mitochondrial membrane potential, pointing to increased induction of apoptosis when compared to doxorubicin alone [22]. Juszczak et al. have shown that 24 h preincubation with retinoic acid and subsequent 24 h incubation with doxorubicin increased the amount of apoptotic HL-60/DOXO cells, compared to doxorubicin alone [16].

### 2.5. Oxidative Stress Evaluation with H_2_DCFDA

**Figure 10:**
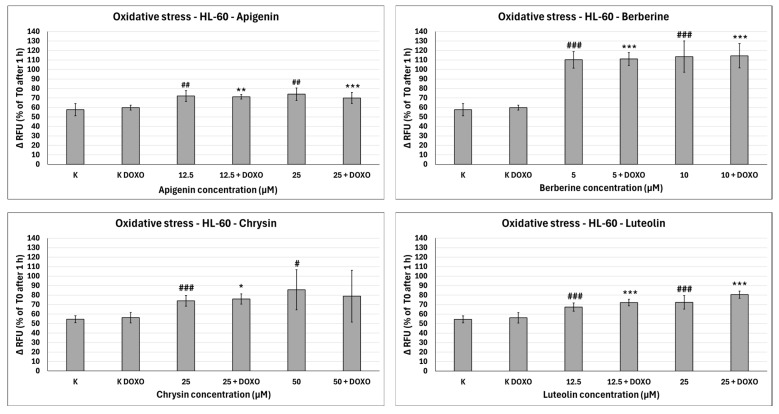
Doxorubicin-induced oxidative stress after 48 h preincubation with ABCL followed by 24 h incubation with doxorubicin. Results are shown as mean percentage ± SD, # *p* < 0.05, ## *p* < 0.01, and ### *p* < 0.001, compared to the control (K), and * *p* < 0.05, ** *p* < 0.01, and *** *p* < 0.001, compared to the respective doxorubicin control (K DOXO). RFU—relative fluorescence unit.

**Figure 11:**
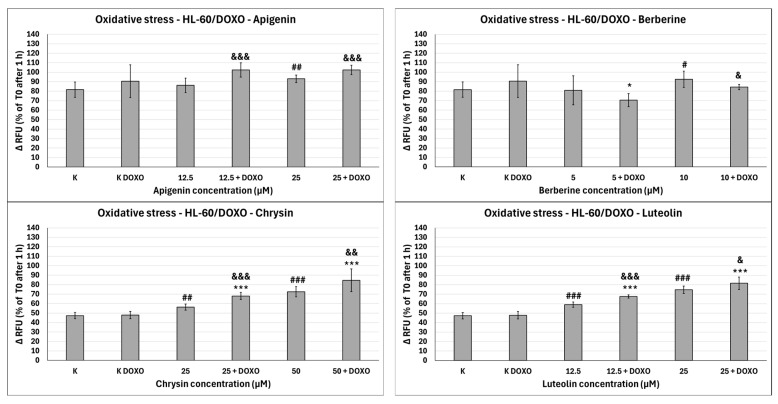
Doxorubicin-induced oxidative stress after 48 h preincubation with ABCL followed by 24 h incubation with doxorubicin. Results are shown as mean percentage ± SD, # *p* < 0.05, ## *p* < 0.01, and ### *p* < 0.001, compared to the control (K), * *p* < 0.05, and *** *p* < 0.001, compared to the respective doxorubicin control (K DOXO), & *p* < 0.05, && *p* < 0.01, and &&& *p* < 0.001, compared to the respective ABCL concentration. RFU—relative fluorescence unit.

ABCL exhibits the ability to increase oxidative stress in both doxorubicin-sensitive HL-60 cells (Figure 10) as well as resistant HL-60/DOXO cells (Figure 11). Oxidative stress-inducing properties of doxorubicin are basically nonexistent in doxorubicin control (K DOXO) (evaluation of doxorubicin’s ability to induce oxidative stress in HL-60/DOXO cells is included in the Supplementary Materials, Figure S4). However, in doxorubicin-resistant cells, ABCL does in fact seem to affect this property of doxorubicin.

In doxorubicin-sensitive HL-60 cells, Apigenin increased relative fluorescence units to 72.10 ± 5.68 at 12.5 µM (*p* < 0.01) and 74.01 ± 6.59 at 25 µM (*p* < 0.01), compared to 57.68 ± 6.46 in control; samples with doxorubicin yielded similar results (Figure 10). In doxorubicin-resistant HL-60/DOXO cells, Apigenin increased relative fluorescence units to 93.04 ± 4.09 at 25 µM (*p* < 0.01) compared to 81.61 ± 8.16 in control (Figure 11).

In doxorubicin-sensitive HL-60 cells, Berberine increased relative fluorescence units to 110.19 ± 8.66 at 5 µM (*p* < 0.001) and 113.61 ± 16.57 at 10 µM (*p* < 0.001), compared to 57.68 ± 6.46 in control; samples with doxorubicin yielded similar results (Figure 10). In doxorubicin-resistant HL-60/DOXO cells, Berberine increased relative fluorescence units to 92.57 ± 8.65 at 10 µM (*p* < 0.05) compared to 57.68 ± 6.46 in the control. A total of 5 µM Berberine combined with doxorubicin lowered relative fluorescence units to 70.50 ± 6.89 (*p* < 0.05), compared to 90.55 ± 17.24 in the doxorubicin control (Figure 11).

In doxorubicin-sensitive HL-60 cells, Chrysin increased relative fluorescence units to 74.01 ± 5.58 at 25 µM (*p* < 0.001) and 85.54 ± 21.14 at 50 µM (*p* < 0.05), compared to 54.56 ± 3.72 in control; samples with doxorubicin yielded similar results (Figure 10). In doxorubicin-resistant HL-60/DOXO cells, Chrysin increased relative fluorescence units to 56.29 ± 3.31 at 25 µM (*p* < 0.01) and 72.44 ± 5.33 at 50 µM (*p* < 0.001), compared to 47.27 ± 3.37 in control (Figure 9). In combination with doxorubicin, Chrysin increased relative fluorescence units to 67.99 ± 3.73 at 25 µM (*p* < 0.001) and 84.59 ± 11.98 at 50 µM (*p* < 0.001), compared to 47.83 ± 3.94 in the doxorubicin control (Figure 11).

In doxorubicin-sensitive HL-60 cells, Luteolin increased relative fluorescence units to 67.41 ± 4.29 at 12.5 µM (*p* < 0.001) and 72.50 ± 7.14 at 25 µM (*p* < 0.001), compared to 54.56 ± 3.72 in control; samples with doxorubicin yielded similar results (Figure 10). In doxorubicin-resistant HL-60/DOXO cells, Luteolin increased relative fluorescence units to 58.97 ± 2.85 at 12.5 µM (*p* < 0.001) and 74.70 ± 3.88 at 25 µM (*p* < 0.001), compared to 47.27 ± 3.37 in the control. In combination with doxorubicin, Luteolin increased relative fluorescence units to 67.59 ± 1.74 at 12.5 µM (*p* < 0.001) and 81.57 ± 6.58 at 25 µM (*p* < 0.001), compared to 47.83 ± 3.94 in the doxorubicin control (Figure 11).

In HL-60/DOXO cells, Apigenin at 12.5 µM combined with doxorubicin increased the amount of relative fluorescence units to 102.38 ± 7.52 (*p* < 0.001) compared to 86.15 ± 7.72 in Apigenin alone, and at 25 µM to 102.33 ± 4.97 (*p* < 0.001) compared to 93.04 ± 4.09 in Apigenin alone (Figure 11). Berberine at 10 µM in combination with doxorubicin lowered relative fluorescence units to 84.28 ± 2.79 (*p* < 0.05) compared to 92.57 ± 8.65 in Berberine alone. Chrysin at 25 µM combined with doxorubicin increased the amount of relative fluorescence units to 67.99 ± 3.73 (*p* < 0.001) compared to 56.29 ± 3.31 in Chrysin alone, and at 50 µM concentration to 84.59 ± 11.98 (*p* < 0.01) compared to 72.44 ± 5.33 in Chrysin alone. Luteolin at 12.5 µM combined with doxorubicin increased the amount of relative fluorescence units to 67.59 ± 1.74 (*p* < 0.001) compared to 58.97 ± 2.85 in Luteolin alone and at 25 µM to 81.57 ± 6.58 (*p* < 0.05) compared to 74.70 ± 3.88 in Luteolin alone (Figure 11).

Overall, ABCL does not seem to affect doxorubicin’s ability to induce oxidative stress in doxorubicin-sensitive HL-60 cells; they themselves, however, caused a slight increase in endogenous ROS, with one exception being Berberine, which doubled the amount of ROS measured in this cell line (Figure 10). In doxorubicin-resistant HL-60/DOXO cells, Apigenin caused a rise in ROS only at a 25 µM concentration; at the same time, it enhanced the oxidative stress-inducing properties of doxorubicin at both 12.5 and 25 µM concentrations (Figure 9). Interestingly, Berberine at 5 µM concentration, when combined with doxorubicin, caused even lower levels of ROS than in the doxorubicin control (*p* < 0.05), which even further hints at its code of conduct being NRF2 activation. Chrysin and Luteolin cause an increase in ROS levels when combined with doxorubicin (*p* < 0.001) (Figure 9).

Another noteworthy thing is that ABCLs are all anti-oxidants; therefore, it is fair to assume that the observed results are the effect of two antagonistic properties of these compounds. On one hand, they act as NRF2 inhibitors, hindering cells’ anti-oxidant responses, and on the other, they themselves act as anti-oxidants. Having all that in mind, it is fair to claim that Apigenin, Chrysin, and Luteolin do hinder cells’ anti-oxidant responses. Similar results with the use of Luteolin were obtained by Yang et al., but in their study, it also increased ROS generation in HT29 cells [24].

Apigenin, Chrysin, and Luteolin exhibited the ability to boost both cytotoxic and DNA-damaging effects of doxorubicin in both doxorubicin-sensitive and doxorubicin-resistant cell lines. Interestingly, while they did not increase in apoptosis levels after doxorubicin treatment in doxorubicin-sensitive cells, they increased in doxorubicin-resistant cells, which may point to the inhibition of NRF2 in this cell line as an underlying mechanism. Their effects on doxorubicin-resistant cells’ DNA damage levels are also interesting; on their own, they generally reduced DNA damage; however, in combination with doxorubicin, their genotoxic properties were greatly increased. Berberine, on the other hand, seems to act more as an NRF2 activator, rather than an inhibitor. While it reduced the percentage of metabolizing cells in combination with doxorubicin in the resazurin assay, after examination of the comet assay and apoptosis assay results, it is much more likely that these deviations are a result of Berberine’s high cytotoxicity towards cancer cells. Apigenin, Chrysin, and Luteolin hinder HL-60 cells’ anti-oxidant responses, and in combination with doxorubicin, they have been shown to significantly enhance its ability to induce oxidative stress, as on its own doxorubicin caused little to no difference. Berberine, when combined with doxorubicin, caused the doxorubicin-resistant cells to strengthen their anti-oxidant response, which even further proves that, in HL-60 cells, Berberine causes NRF2 activation.

Other studies have found that Luteolin inhibits the NRF2 pathway in vivo using C57BL/6 mice, where it greatly reduced the expression of NRF2-controlled genes like *NQO1*, *AKR1C*, *HO-1*, and *GSTm1* [25]. On top of that, it was shown that Luteolin alone, as well as in combination with cisplatin, in xenograft mice inoculated with human non-small lung cancer A549 cells, greatly reduced the tumor growth; furthermore, analysis of tumors after Luteolin treatment also revealed lowered expression of NRF2-controlled proteins. Lowered GSH levels were also noted after Luteolin treatment [25]. Another in vivo study, on the other hand, mentions luteolin acting as an NRF2 activator in mice liver, promoting its nuclear translocation, which led to increased expression of *HO-1* and *NQO1* genes [26]. In another study, it was found that Luteolin sensitized two oxaliplatin-resistant colorectal cancer cell lines, namely HCT116-OX and SW620-OX, both of which have shown lowered protein levels of NRF2 and NQO1 in vitro after Luteolin treatment. Further studies on xenograft mice have shown that treatment with Luteolin caused lowered expression of NQO1, HO-1, and GSTα1/2, as well as lowered levels of GSH. Luteolin also induced higher sensitivity to doxorubicin, cisplatin, and oxaliplatin in those two cell lines [27]. In yet another study, Luteolin treatment reduced expression of NRF2, HO-1, Cripto-1, and Sirt3 protein levels in MDA-MB-231 breast cancer cells [28]. Luteolin was also shown to reduce mRNA expression of *HO-1*, *NQO1*, *GCLC*, *MRP2*, and *AKR1C1* in an MCF7 breast cancer cell line through increasing KEAP1-independent NRF2 mRNA degradation, as well as reducing GSH levels in A549 cells and sensitizing them to anti-cancer drugs like bleomycin, oxaliplatin, and doxorubicin [29]. Chrysin was able to sensitize BEL-7402/ADM and MCF/ADM cells to doxorubicin. In BEL-7402/ADM, a decrease in mRNA levels of NRF2 was observed, as well as of its target genes *HO-1*, *AK1B10*, and *MRP5* [30]. Berberine was successfully used in surmounting lapatinib resistance in BT-474^LapR^ and AU-565^LapR^ breast cancer cells. It has caused lowered viability of the cells and elevated apoptosis induction, as well as increased induction of ROS when combined with lapatinib. This was proven to be the result of NRF2 inhibition through KEAP1-independent ubiquitination of the NRF2 protein [31].

## 3. Materials and Methods

### 3.1. Chemicals

Doxorubicin hydrochloride (doxorubicin), 2′,7′-dichlorofluorescein diacetate (H_2_DCF-DA), Hank’s balanced salt solution (HBSS), dimethyl sulfoxide (DMSO), and hydrogen peroxide (H_2_O_2_) were purchased from Sigma-Aldrich (St. Louis, MO, USA). All other chemicals were of the highest commercial grade available. ABCL were dissolved in DMSO, acquiring working solutions at a 10 mM concentration. The stock solution of doxorubicin (1 mM) was dissolved in DNase/RNase-free water.

### 3.2. Cell Culture

The HL-60 (human promyelocytic leukemia) cell line was obtained from the American Type Culture Collection (ATCC) and cultured in Iscove’s Modified Dulbecco’s Medium (IMDM) with 15% fetal bovine serum (FBS), 2 mM L-Glutamine, 25 mM HEPES, and penicillin/streptomycin solution (100 U/mL and 100 µg/mL, respectively). The cell line was cultured in flasks at 37 °C in 5% CO_2_ and sub-cultured every 2–3 days to maintain exponential growth.

HL-60/DOXO cell line was derived from the HL-60 cell line by long-term exposure to a continuous stepwise increase in doxorubicin concentration, as described in Juszczak et al. [16]. This cell line has been confirmed to have elevated expression of NRF2 at both protein and mRNA levels [16].

### 3.3. Cell Viability and Experimental Schemes

#### 3.3.1. Cell Viability Resazurin Assay

The cell viability resazurin assay was conducted based on the method described by O’Brien et al. [32]. HL-60 and HL-60/DOXO cells were incubated in 96-well plates for a set amount of time—either 24 h or 48 h. After incubation, 10 μL of resazurin salt (2 mg/10 mL PBS) was added to each well. Fluorescence was measured with a microplate reader Synergy HT (Bio-Tek Instruments, Winooski, VT, USA), using an excitation wavelength of 530/25 and an emission wavelength of 590/35 nm; after that, plates were again incubated at 37 °C in 5% CO_2_ for 2 h. After that, the time measurement was repeated using the same parameters. The effects of compounds were calculated as the percentage of control fluorescence. All assays were performed in octuplicate.

##### ABCL Cytotoxicity

HL-60 and HL-60/DOXO cells were seeded in 96-well plates at a count of 1 × 10^4^ per well. ABCL was added to wells to obtain final concentrations of 0.390625, 0.78125, 1.5625, 3.125, 6.25, 12.5, 25, and 50 μM, and the total volume per well was 100 μL. Plates were then incubated at 37 °C in 5% CO_2_ for 24 h and 48 h.

##### Preincubation Scheme with ABCL

HL-60 and HL-60/DOXO cells were seeded onto Petri dishes at a count of 3 × 10^5^ per Petri dish. ABCL were added to achieve the following final concentrations: Apigenin—5, 10, 25 μM; Berberine—3.7, 7.5, 15 μM for 24 h incubation and 2.5, 5, and 10 μM for 48 h incubation; Chrysin—10, 25, 50 μM; and Luteolin—5, 10, 25 μM; and the total volume per Petri dish was 3 mL. Petri dishes were incubated at 37 °C in 5% CO_2_ for 24 h and 48 h. After that time, the cells were collected and centrifuged in an Eppendorf Centrifuge 5403 at 1000 rpm at 23 °C for 5 min, and the supernatant was removed and the cells were washed with sterile PBS, suspended in fresh medium, and seeded in 96-well plates at a count of 1 × 10^4^ per well. DOXO was added to the wells to obtain a final concentration of 116 nM for the doxorubicin-sensitive line and 556 nM for the doxorubicin-resistant line; the total volume per well was 100 μL. Plates were then incubated at 37 °C in 5% CO_2_ for 24 h.

##### Coincubation Scheme with ABCL

HL-60 and HL-60/DOXO cells were seeded in 96-well plates at a count of 1 × 10^4^ per well. Compounds were added to the wells to obtain final concentrations of Apigenin—5, 10, 25 μM, Berberine—3.7, 7.5, 15 μM, Chrysin—2.5, 5, 10 μM, and Luteolin—5, 10, 25 μM. Doxorubicin was also added to the wells to obtain a final concentration of 116 nM for the doxorubicin-sensitive cell line and 556 nM for the doxorubicin-resistant cell line (equal to approximate IC_50_ values for each respective cell line); the total volume per well was 100 μL. Plates were then incubated at 37 °C in 5% CO_2_ for 24 h.

### 3.4. DNA Damage Evaluation—The Comet Assay

HL-60 and HL-60/DOXO cells were seeded onto Petri dishes at a count of 3 × 10^5^ per Petri dish. ABCL was added to achieve final concentrations of Apigenin—12.5 and 25 μM, Berberine—5 and 10 μM, Chrysin—25 and 50 μM, and Luteolin—12.5 and 25 μM; the total volume per Petri dish was 3 mL. Petri dishes were incubated at 37 °C in 5% CO_2_ for 48 h. After that time, cells were collected and centrifuged in an Eppendorf Centrifuge 5403 at 1000 rpm at 23 °C for 5 min, supernatant was removed, and cells were washed with sterile PBS, after which they were suspended in fresh medium and seeded on new Petri dishes. Doxorubicin was added to the Petri dishes to obtain a final concentration of 58 nM for the doxorubicin-sensitive line and 278 nM for the doxorubicin-resistant line (half of the respective IC_50_ values, and the concentration was lowered to ensure clarity of results); the total volume per Petri dish was 3 mL. Petri dishes were then incubated at 37 °C in 5% CO_2_ for 24 h. After that time, cells were collected and counted in the Bürker chamber, onto which a mixture of 10 μL of cell suspension and 10 μL trypan blue was added. Counting was performed under an inverted light microscope NIB 100 at 10 × 20 magnification. A total of 50,000 cells were taken from each sample and suspended in 0.5 mL of fresh medium. All samples were then centrifuged in the MPW-260R centrifuge by MPW with REF 11,461 rotor at 1700 rpm for 7 min. A total of 450 μL of supernatant was removed from the samples, and 40 μL of 0.75% LMP agarose was added. After thorough mixing, 40 μL of the mixture was added onto microscope slides, which were pre-coated with 0.5% NMP agarose twice per slide, and covered with cover slips. Slides were transferred onto a cold table. After agarose had set, the cover slips were carefully removed. Slides were then placed in an ice-cold lysing buffer (2.5 M NaCl, 100 mM EDTA, 10 mM Tris, 1% Triton X-100, pH = 10). Lysis lasted for 1 h at 4 °C. After that time, the lysing buffer was removed and slides were rinsed with ice-cold developing buffer (300 mM NaOH, 1 mM EDTA, pH > 13) 3 times and then suspended in it for 20 min at 4 °C. After that time, the slides were rinsed with electrophoretic buffer (30 mM NaOH, 1 mM EDTA, pH > 13) 3 times. Slides were placed in an electrophoresis apparatus with an MP-300V power supply by Biocom. Slides were tightly placed, and empty slides were placed at the periphery of the apparatus. Electrophoresis was conducted at 17 V (0.73 V/cm) and 32 mA for 20 min. After electrophoresis, slides were rinsed with distilled water 3 times and stained with 40 μL of DAPI (4 μg/mL) for 1 h in complete darkness. After that time, the slides were observed under a fluorescence microscope, Eclipse E400 by Nikon (Tokyo, Japan), at 10 × 20 magnification with the use of an integrated ProgRes MF cool monochrome video camera (JENOPTIK, Jena, Germany). From each sample, 100 cells were evaluated using Lucia Comet Assay version 7.30 (Laboratory Imaging, Praha, Czech Republic) with the following parameters: exposure: 200 ms, gain: 5.0, offset: 3.0, and gamma: 0.80. The percentage of DNA in the comet tail was used as an indicator of DNA damage. The comet assay was performed according to the procedure described by Singh et al. [33].

### 3.5. Annexin V and Propidium Iodide Apoptosis Assay

HL-60 and HL-60/DOXO cells were seeded onto Petri dishes at a count of 3 × 10^5^ per Petri dish. ABCL was added to achieve final concentrations of Apigenin—25 μM, Berberine—10 μM, Chrysin—25 μM, and Luteolin—25 μM; the total volume per Petri dish was 3 mL. Petri dishes were incubated at 37 °C in 5% CO_2_ for 48 h. After that time, cells were collected and centrifuged in an Eppendorf Centrifuge 5403 at 1000 rpm at 23 °C for 5 min; the supernatant was removed, and cells were washed with sterile PBS, and then they were suspended in fresh medium and seeded on new Petri dishes. Doxorubicin was added to the Petri dishes to obtain a final concentration of 116 nM for both doxorubicin-sensitive and doxorubicin-resistant lines (the lower concentration used for the doxorubicin-resistant cell line is due to the fact that doxorubicin and propidium iodide have similar excitation wavelengths and thus higher concentrations, rendering the results unreadable); the total volume per Petri dish was 3 mL. Petri dishes were then incubated at 37 °C in 5% CO_2_ for 24 h. After that time, cells were collected and centrifuged in an Eppendorf Centrifuge 5403 at 1000 rpm at 23 °C for 5 min, supernatant was removed, and cells were washed twice with cold PBS. Propidium iodide (PI) and annexin V (FITC) controls were suspended in 1 mL of cold PBS; next, the samples were placed on a vortex and 1 mL of 96% ethanol at −20 °C was slowly added, after which samples were aerated using a shepherd’s pipette and centrifuged in MPW-260R by MPW with REF 11,461 rotor, at 3000 rpm for 10 min. Supernatant was removed, and cells were suspended in 300 μL of binding buffer with 3 μL of PI or FITC, respectively. Remaining samples were suspended in a mixture of 300 μL of binding buffer with 3 μL of PI and 3 μL of FITC. All samples were incubated for 15 min at 25 °C in complete darkness. After that time, samples were placed on ice. Samples were measured using an LS.RII flow cytometer (Becton Dickinson, San Jose, CA, USA).

### 3.6. Intracellular ROS Assay with H_2_DCFDA

HL-60 and HL-60/DOXO cells were seeded onto Petri dishes at a count of 3 × 10^6^ per Petri dish. Compounds were added to achieve final concentrations of Apigenin—12.5 and 25 μM, Berberine—5 and 10 μM, Chrysin—25 and 50 μM, and Luteolin—12.5 and 25 μM; the total volume per Petri dish was 10 mL. Petri dishes were incubated at 37 °C in 5% CO_2_ for 48 h. Cells were then washed with PBS, suspended in fresh medium, seeded onto new Petri dishes with the addition of doxorubicin, achieving a final concentration of 20 nM, and then incubated for another 48 h. Cells were then washed twice with warm HBSS. Out of each sample, 1.6 × 10^6^ cells were taken, and 2′,7′-dichlorofluorescein diacetate (H_2_DCFDA) was added up to a final concentration of 20 μM. Cells were then incubated for 30 min in the dark at 37 °C. After that time, cells were centrifuged at 3000 rpm at 23 °C for 5 min. Supernatant was removed, and cells were washed twice with warm HBSS. Cells were suspended in warm HBSS and seeded in a black 96-well plate at a count of 1 × 10^5^ per well, total volume per well was 100 μL. Fluorescence was measured with a microplate reader Synergy HT (Bio-Tek Instruments, Winooski, VT, USA, using an excitation wavelength of 495/25 and an emission wavelength of 520/35 nm directly after seeding and then after 15, 30, 45, and 60 min.

### 3.7. Data Analysis

Statistical significance was calculated with Statistica (ver. 14.1.0.4), using a *t*-test for dependent samples. Cell viability assay data, apoptosis assay data, and oxidative stress evaluation data are presented as mean ± SD (standard deviation). Comet assay data are presented as mean ± SEM (standard error mean). The differences were considered statistically significant when the *p*-value was <0.05 at maximum.

IC_50_ was calculated using the AAT Bioquest IC_50_ calculator (https://www.aatbio.com/tools/ic50-calculator (accessed on 22 October 2025).

## 4. Conclusions

The findings demonstrate that Apigenin, Chrysin, and Luteolin possess substantial potential to overcome doxorubicin resistance in HL-60 leukemic cells. In contrast, the effects of Berberine remain inconclusive due to considerable variability reported across studies.

## Data Availability

The original contributions presented in this study are included in the article/Supplementary Materials. Further inquiries can be directed to the corresponding author.

## Supplementary Materials

The following supporting information can be downloaded at https://www.mdpi.com/article/10.3390/ijms262110565/s1.
