# Supplementary material for: Potential of Apigenin, Berberine, Chrysin, and Luteolin to Overcome Doxorubicin Resistance in Acute Promyelocytic Leukemia HL-60 Cells
Source: Int J Mol Sci. 2025 Oct 30;26(21):10565. doi: 10.3390/ijms262110565 (PMC12610337; doi:10.3390/ijms262110565)
Supplement: Supplementary file 1 [file ijms-26-10565-s001.zip › ijms-3883563-SI.pdf]

Supplementary Materials

Potential of Apigenin, Berberine, Chrysin, and Luteolin to Overcome Doxorubicin Resistance in Acute Promyelocytic Leukemia HL-60 Cells

Piotr Wadowski[1,2] and Katarzyna Woźniak[1]*

[1] University of Lodz, Faculty of Biology and Environmental Protection, Department of Molecular Genetics, Pomorska 141/143, 90-236, Lodz, Poland

[2] Doctoral School of Exact and Natural Sciences, University of Lodz, Banacha 12/16, 90-237 Lodz, Poland

* Correspondence: Katarzyna Woźniak, e-mail: katarzyna.wozniak@biol.uni.lodz.pl

Tel.: +48-42-635-47-76; Fax: +48-42-635-44-84

1. ABCL cytotoxicity

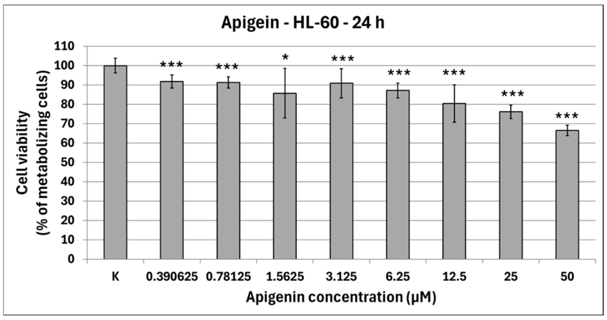

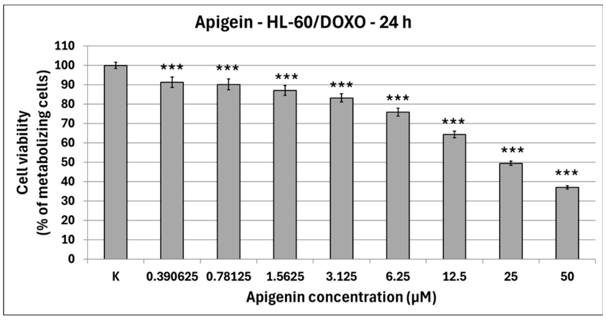

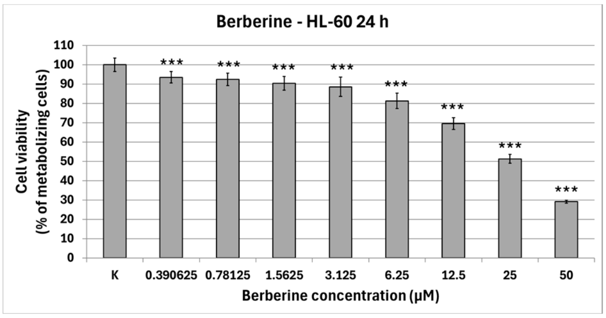

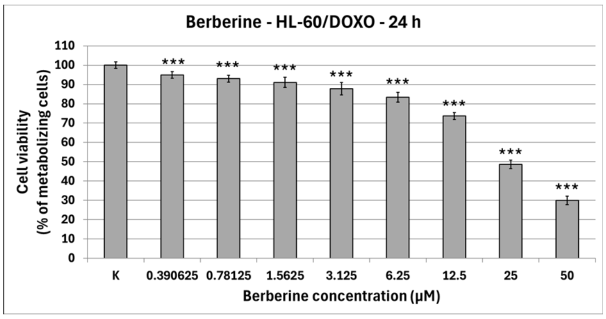

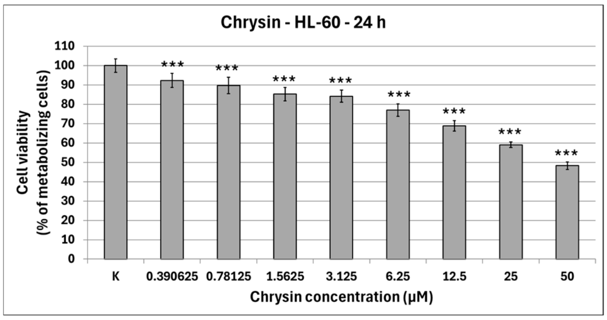

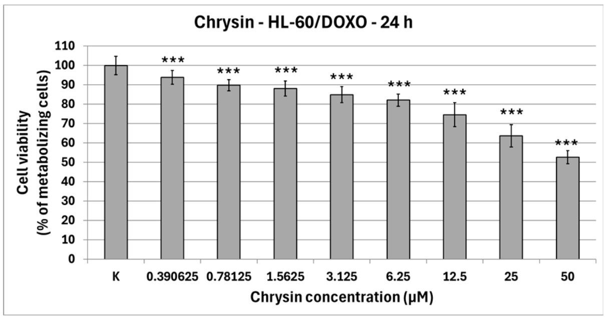

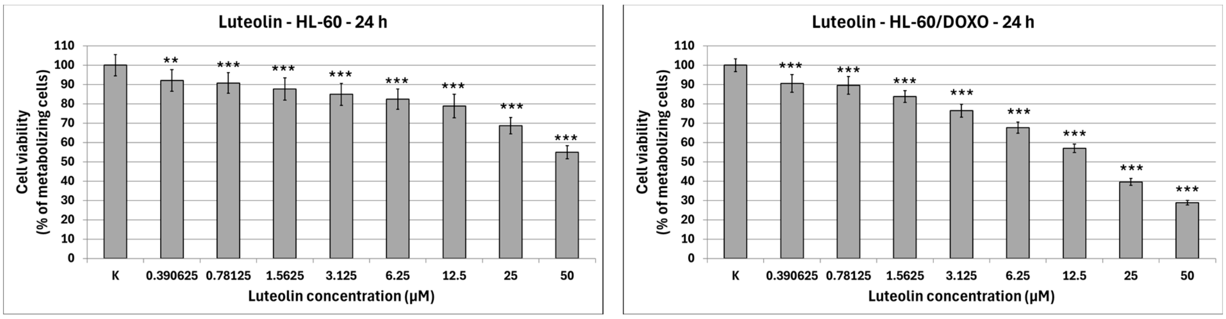

Figure S1. ABCL cytotoxicity after 24 h incubation in HL-60 and HL-60/DOXO cell lines. Results are shown as mean percentage ± SD, * $p < 0.05$. ** $p < 0.01$, *** $p < 0.001$, compared to the control (K).

2. 24 h Preincubation scheme with ABCL

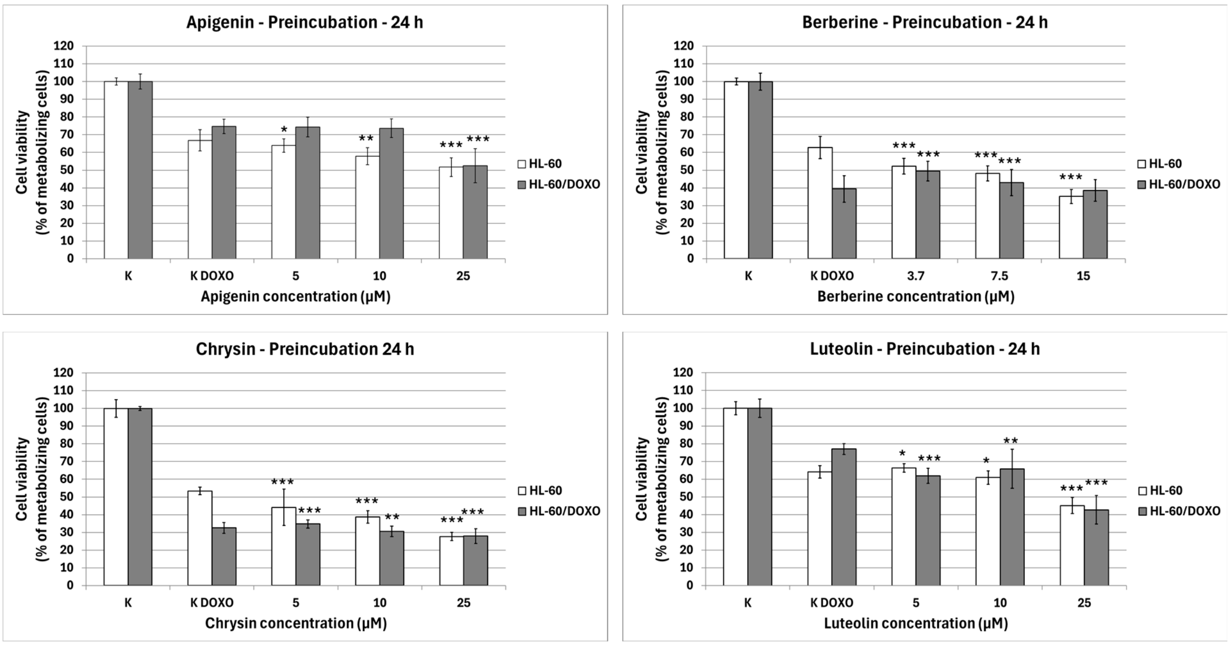

Figure S2. Doxorubicin cytotoxicity after 24 h preincubation with ABCL followed by 24 h incubation with doxorubicin. Results are shown as mean percentage ± SD, * $p < 0.05$. ** $p < 0.01$, *** $p < 0.001$, compared to the doxorubicin control (K DOXO).

3. 24 h Coincubation scheme with ABCL

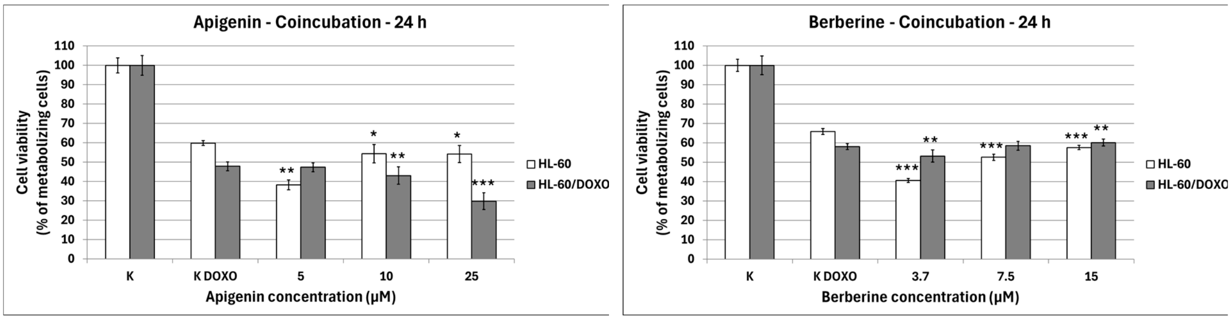

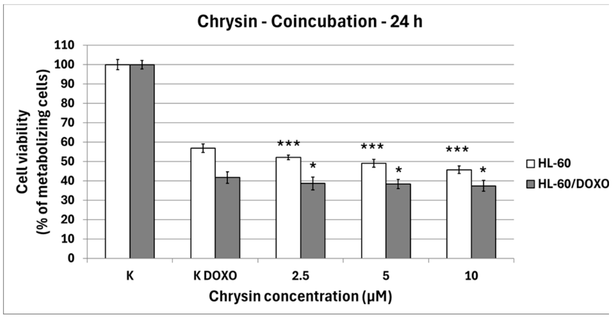

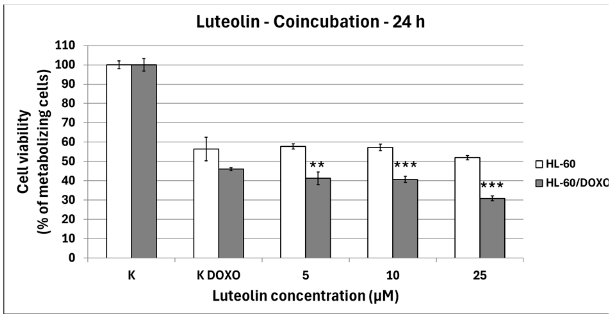

Figure S3. Doxorubicin cytotoxicity after 24 h of coincubation with ABCL followed by 24 h incubation with doxorubicin. Results are shown as mean percentage ± SD, * $p < 0.05$. ** $p < 0.01$, *** $p < 0.001$, compared to the doxorubicin control (K DOXO).

4. Oxidative stress – doxorubicin – HL-60/DOXO cells

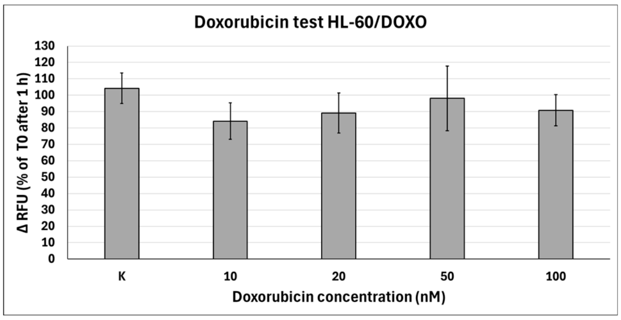

Figure S4. Doxorubicin-induced oxidative stress after 24 h incubation of HL-60/DOXO cells. Results are shown as mean percentage ± SD, $p > 0.05$, compared to the control (K).
